# Supplementary material for: Risk factors for asthma among schoolchildren who participated in a case-control study in urban Uganda
Source: eLife. 2019 Nov 15;8:e49496. doi: 10.7554/eLife.49496 (PMC6914334; doi:10.7554/eLife.49496)
Supplement: Supplementary file 1. — Supplementary file 1a The association between infection with different species of helminths and asthma among 1543 schoolchildren. Three fresh stool samples per child were examined for helminths using the Kato Katz method. We used multiple logistic regression method, adjusted for child’s age, sex, residence at birth, father’s education level and reported worm treatment in the last 12 months. ¤Other helminth infections included Hymenolepis nana and Enterobius vermicularis. N = number; Adj. OR = adjusted odds ratio; CI = confidence interval; number (%) in 2nd and 3rd column. Supplementary file 1b The association between individual and combined effects of child’s atopy and parental history of allergic disease, and asthma risk among 1501 schoolchildren. Skin prick test (SPT) performed using standard procedures and seven crude extracts of Blomia tropicalis, Dermatophagoides mix, cockroach, peanut, cat, weeds pollen mix, and mould mix. Parental history of allergic disease included a history of asthma, eczema, allergic rhinitis, allergic conjunctivitis and any other allergies. We used multiple logistic regression analysis, and adjusted for child’s age, sex, residence at birth and father’s education level. N = number; Adj. OR = adjusted odds ratio; CI = confidence interval; ‘-' refers to none, ‘+' refers to present. Number (%) in 3rd and 4th column. Supplementary file 1c The individual and combined effects of mother’s and father’s history of allergic disease, and asthma risk among 1498 schoolchildren. Parental history of allergic disease included a history of asthma, eczema, allergic rhinitis, allergic conjunctivitis and any other allergies. We conducted multiple logistic regression analysis, and adjusted for child’s age, sex, residence at birth and father’s education level. N = number; Adj. OR = adjusted odds ratio; CI = confidence interval; number (%) shown in 2nd and 3rdcolumn. [file elife-49496-supp1.docx]

**Supplementary file 1a:** **Helminth infections among schoolchildren enrolled in an asthma case-control study between 2015-17 in Uganda (N=1,543)**

| **Type of helminth infection** | **Asthma cases**  **N=513** | **Non-asthma controls**  **N=1,030** | **Adj. OR (95% CI)** |
| --- | --- | --- | --- |
| **Any helminth infection** | | | |
| None (1,337) | 460 (89.7) | 877 (85.1) | 1 |
| Yes (206) | 53 (10.3) | 153 (14.9) | 0.75 (0.53-1.07) |
| ***Schistosoma mansoni*** | | | |
| None (1,414) | 477 (93.0) | 937 (91.0) | 1 |
| Yes (129) | 36 (7.0) | 93 (9.0) | 0.81 (0.53-1.23) |
| ***Trichuris trichiura*** | | | |
| None (1,506) | 508 (99.0) | 998 (96.9) | 1 |
| Yes (37) | 5 (1.0) | 32 (3.1) | 0.33 (0.13-0.89) |
| **Hookworm** | | | |
| None (1,524) | 510 (99.4) | 1,014 (98.5) | 1 |
| Yes (19) | 3 (0.6) | 16 (1.5) | 0.59 (0.16-2.18) |
| ***Ascaris lumbricoides*** | | | |
| None (1,537) | 512 (99.8) | 1,025 (99.5) | 1 |
| Yes (6) | 1 (0.2) | 5 (0.5) | 0.75 (0.08-6.82) |
| **Other helminth infections**^¤^ | | | |
| None (1,507) | 501 (97.7) | 1,006 (97.7) | 1 |
| Yes (36) | 12 (2.3) | 24 (2.3) | 1.36 (0.64-2.89) |

**Supplementary file 1b:** **Combined effects of atopy and parental history of allergic disease as asthma risk factors among schoolchildren enrolled in a case-control study in Uganda from 2015-17 (N=1,501)**

| **Atopy (SPT)** | **Parental history of allergy** | **Asthma cases N=518** | **Non-asthma controls N=983** | **Adj. OR (95% CI)** |
| --- | --- | --- | --- | --- |
| - | - | 77 (14.9) | 394 (40.1) | 1 |
| + | - | 105 (20.3) | 168 (17.1) | 3.07 (2.14-4.41) |
| - | + | 149 (28.7) | 272 (27.7) | 2.79 (2.01-3.87) |
| + | + | 187 (36.1) | 149 (15.1) | 5.74 (4.07-8.10) |

**Supplementary file 1c: Combined effects of maternal and paternal history of allergic disease as asthma risk factors among schoolchildren in a case-control study in Uganda between 2015-17 (N=1,498)**

| **Parental reported history of allergic disease** | **Asthma cases N=511** | **Non-asthma controls N=987** | **Adj. OR (95% CI)** |
| --- | --- | --- | --- |
| Neither parent | 184 (36.0) | 572 (58.0) | 1 |
| Mother only | 159 (31.1) | 232 (23.5) | 2.15 (1.63-2.83) |
| Father only | 89 (17.4) | 87 (8.8) | 2.74 (1.92-3.92) |
| Both parents | 79 (15.5) | 96 (9.7) | 2.49 (1.74-3.58) |
